# Supplementary figures and images for: Degradation of lignocelluloses in straw using AC-1, a thermophilic composite microbial system
Source: PeerJ. 2021 Nov 1;9:e12364. doi: 10.7717/peerj.12364 (PMC8567851; doi:10.7717/peerj.12364)

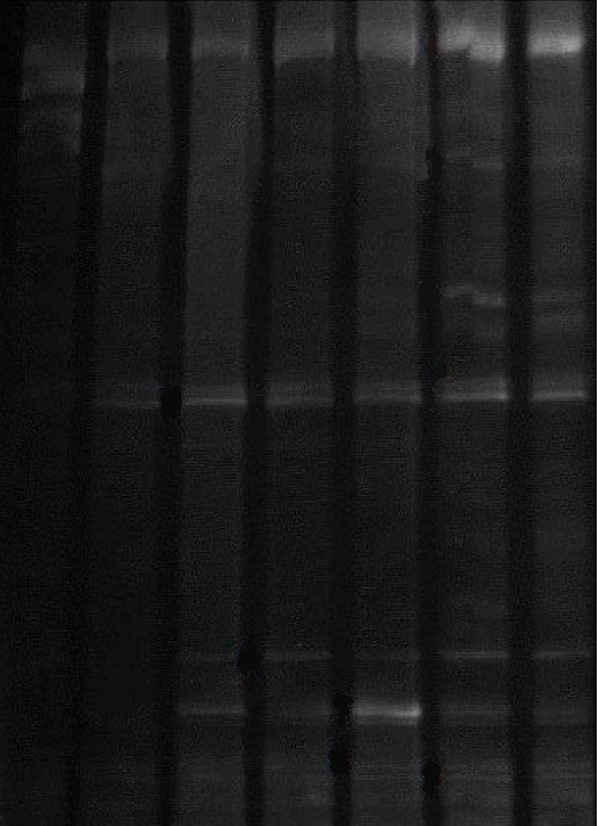

Supplement: Supplemental Information 2 [file peerj-09-12364-s002.zip › Fig 4 - ╕▒▒╛.jpg]
